# Impact assessment of high soil CO_2_ on plant growth and soil environment: a greenhouse study

**DOI:** 10.7717/peerj.6311

**Published:** 2019-01-25

**Authors:** Wenmei He, Gayoung Yoo, Mohammad Moonis, Youjin Kim, Xuanlin Chen

**Affiliations:** Department of Applied Environmental Science, Kyung Hee University, Yongin-si, South Korea

**Keywords:** CO_2_ stress, High soil CO_2_, Carbon capture and storage, Root water absorption activity, Plant response, Impact assessment, O_2_ depletion

## Abstract

To ensure the safety of carbon capture and storage (CCS) technology, insight into the potential impacts of CO_2_ leakage on the ecosystem is necessary. We conducted a greenhouse experiment to investigate the effects of high soil CO_2_ on plant growth and the soil environment. Treatments comprised 99.99% CO_2_ injection (CG), 99.99% N_2_injection (NG), and no injection (BG). NG treatment was employed to differentiate the effects of O_2_ depletion from those of CO_2_ enrichment. Soil CO_2_ and O_2_ concentrations were maintained at an average of 53% and 11%, respectively, under CG treatment. We verified that high soil CO_2_ had negative effects on root water absorption, chlorophyll, starch content and total biomass. Soil microbial acid phosphatase activity was affected by CG treatment. These negative effects were attributed to high soil CO_2_ instead of low O_2_ or low pH. Our results indicate that high soil CO_2_ affected the root system, which in turn triggered further changes in aboveground plant tissues and rhizospheric soil water conditions. A conceptual diagram of CO_2_ toxicity to plants and soil is suggested to act as a useful guideline for impact assessment of CCS technology.

## Introduction

Carbon capture and storage (CCS) technology is considered an important strategy for mitigating climate change (IPCC, 2014). To ensure safe and successful CCS projects, the European Union has published guidance documents for its CCS Directive that include general CO_2_ leakage scenarios (European Communities, 2011; Pearce et al., 2014). The most relevant CO_2_leakage could occur in storage reservoirs via faults, fractures or boreholes (Pearce et al., 2014; Vrålstad et al., 2018). As CO_2_ would likely be transported to storage sites through long pipelines, leakage could also occur via corrosion and connection failures of shallow pipes (European Communities, 2011; Fu & Gundersen, 2012; Pearce et al., 2014). The leaked CO_2_ would diffuse from deep soil layers toward the surface. As a result, soil could experience high CO_2_ concentrations between 40% and 95% for a period of time (Beaubien et al., 2008; Al-Traboulsi et al., 2012; Lake et al., 2013; Paulley et al., 2013). Surrounding plants and soil microbes could be influenced by high concentration of soil CO_2_ and the concomitant low pH and reduced proportions of O_2_ andN_2_.

Plant responses to a high-CO_2_ soil environment have been widely investigated in natural analogues and at artificial CO_2_ release sites (Pfanz et al., 2004; Beaubien et al., 2008; Male et al., 2010; Krüger et al., 2011; Patil, 2012; West et al., 2015). Chlorosis and discoloration were observed in natural vegetation after 4 days of CO_2_ exposure at the Zero Emission Research and Technology Center in the US (Lakkaraju et al., 2010; Sharma et al., 2014). Plant physiological indicators such as chlorophyll content, photosynthesis rate, stomata conductance and transpiration rate were lower in soils with a high CO_2_ concentration (Spangler et al., 2009; Wu et al., 2014). Morphological indicators such as plant height, root length, leaf number, leaf area, seed number and pod number were also reduced after the soil was exposed to high levels of CO_2_ compared with non-gassed controls. Plant biomass also decreased on CO_2_ gassing (Al-Traboulsi et al., 2012; Al-Traboulsi et al., 2013; Wu et al., 2014).

Although previous studies consistently reported that plants exposed to high soil CO_2_ showed inhibited growth (Patil, Colls & Steven, 2010; Al-Traboulsi et al., 2013; Zhao et al., 2017), few clearly identified the main driving factor of these negative impacts (Pfanz et al., 2004; Pfanz & Saßmannshausen, 2008; He et al., 2016). Beaubien et al. (2008) and Stephens & Hering (2002) observed that water absorption of plants was lower, but they did not distinguish the effects of high soil CO_2_ (∼100% at 20 cm depth) from those of low O_2_ or other trace gases such as H_2_S and CH_4_. Al-Traboulsi et al. (2012) and Zhang et al. (2016) explained that reduced bean and maize metabolism in CO_2_ gassing plots might be due to reduced O_2_ level rather than pH changes induced by high soil CO_2_ concentration (50–70% at 15–30 cm depth). Lake et al. (2016a) sought to distinguish the effects of elevated CO_2_ (42.3%) from those of O_2_ depletion (11.1%) on the growth of wheat and beetroot by employing an N_2_ gassing treatment. They suggested that high soil CO_2_ might explain the biomass reduction of beetroots better than O_2_ deficiency. Similar results were found in earlier studies by Chang & Loomis (1945) and Kramer (1940). In their studies, transpiration and root water absorption of wheat, maize, rice, sunflowers and tomatoes were close to normal under O_2_-free nitrogen-bubbling treatment, while they were severely affected by 100% CO_2_ bubbling. Chang & Loomis (1945) also compared the effects of low pH solution and high CO_2_ exposure on root function and observed that toxic effects were more evident under high CO_2_ conditions than under acidic solutions. They suggested that CO_2_ toxicity is a more important factor in plant growth than O_2_ deficiency. Previous studies have implied that high concentration of soil CO_2_ itself might be toxic to plant growth in a variety of plant species. However, compared with the intensive studies of CO_2_ effects on plants in hydroponic system, the assumption of CO_2_ toxicity on plant growth under high soil CO_2_ condition has not been sufficiently proved.

To identify the main factor in soil CO_2_ enrichment that influences plant growth and soil, we conducted a greenhouse experiment using a specially designed pot in which soil CO_2_ was enriched without a buildup of ambient atmospheric CO_2_. We separated the effects of high soil CO_2_ and those of low soil O_2_ by employing an N_2_ gassing treatment. The pot was large enough to grow woody plants, and the injection time covered the main growing period. The objective of this study was to investigate the effects of high soil CO_2_ on plant physiological reactions and their interactions with the soil environment. A conceptual diagram of the effects of CO_2_ toxicity on plant metabolism is proposed for further insight into the impacts of high soil CO_2_.

## Materials & Methods

### Soil and plant preparation

Soils used for incubation included commercial potting soil (Korea Association of Seedbed Media, Seoul, South Korea) and mineral soil procured from a construction company (Seung Hwa Construction, South Korea). Basic soil physicochemical properties and components are summarized in Table 1. Soil pH was measured using a 1:5 dilution method (Thomas, 1996). Total carbon (TC) and total nitrogen (TN) contents were analyzed using combustion analysis with a Carlo Erba NS 1500 C/N analyzer (Carlo Erba, Milan, Italy). A two-year-old crimson glory vine (*Vitis coignetiae*) was selected for this experiment and bought locally (West Suwon Agricultural Products Inc., South Korea). This plant grows well in neutral to alkaline soils and is native to East Asian countries such as Japan and South Korea.

**Table 1:** Physicochemical properties of the soil.

**Soil**	**pH**	**TN**	**TC**	**Composition (%)**					
		(g kg^−1^ soil)		**Zeolite**	**Pearlite**	**Vermiculite**	**Coco peat**	**Peat moss**	**Other**
**Potting**	6.87	6.30	390.32	4.0	7.0	6.0	68.0	14.7	0.3
				**Clay**	**Silt**	**Sand**			
**Mineral**	6.80	0.19	3.02	4.0	26.0	70.0			

### Experimental design

**Figure 1:**
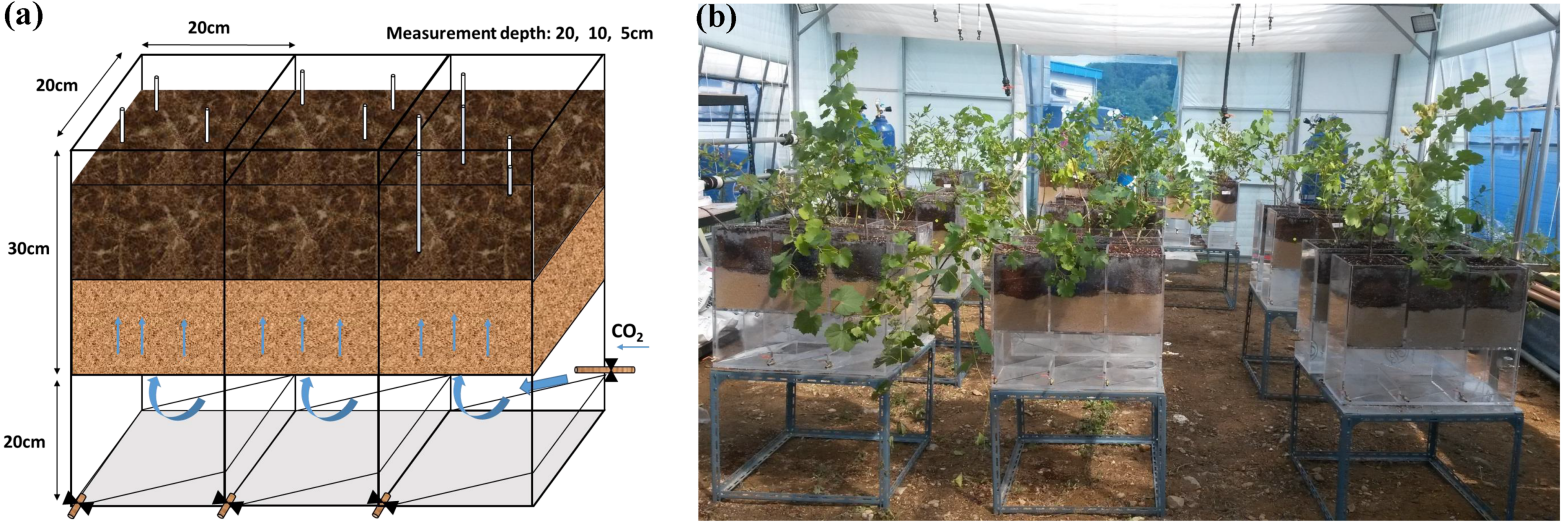
Injection box (A) and treatment layout in the greenhouse (B). Photo credit: Wenmei He.

Gas treatments were applied in a greenhouse on the campus of Kyung Hee University (Yongin-si, South Korea) for 32 days from August 20 to September 23, 2015. Mean temperature was 28 ± 4 °C during the day and 18 ± 5 °C at night. An acrylic container including three pots (30 × 20 × 20 cm; height × length × width) and an empty chamber (20 × 20 × 60 cm; height × length × width) were specially designed (Fig. 1A). At the bottom of each pot, nine drainage holes were covered with mesh for optimal gas diffusion. An empty chamber was connected to a gas tank through a valve. Each pot was filled with 7 kg of mineral soil up to 15 cm from the bottom, and then another 15 cm of potting soil was added. Grape saplings were transplanted on July 15 and adjusted to a new environment for 36 days until August 20. Soil water content in all treatments was adjusted to 20–30% (v/v) and maintained by manual watering every 4 days. In the CG treatment, 99.99% CO_2_ gas was supplied at a rate of 400 mL/min^−1^. The NG treatment was set up using 99.99% N_2_ gas at the same injection rate to investigate the effects of O_2_ depletion without CO_2_ enrichment. The BG treatment was prepared as a control with no injection. Three silicon tubes (2 mm in diameter) with one end connected to a copper fitting covered with mesh (100 µm) were buried in the soil at depths of 5, 10 and 20 cm to measure the CO_2_ and O_2_ concentrations. Although the pot was not large enough to permit lateral heterogeneity of the gases, the tubes at each depth in the replicated pots were located randomly to ensure measurements were representative. Nine replicates were set up for each treatment. The greenhouse remained open and two fans were used to prevent ambient CO_2_ accumulation (Fig. 1B).

### Soil measurements

Daily measurements of soil CO_2_ and O_2_ (% of total 100% by volume) at three depths (5, 10, and 20 cm) were performed using a portable gas detector (COMBI-R, Status Scientific Controls Ltd., Mansfield, Nottinghamshire, UK). The detection range of CO_2_ was from 0 to 100% with an accuracy of ±1% and that of O_2_ was from 0 to 25% with an accuracy of ±0.1%. Soil pH and temperature were measured directly using a pH meter (Field Scout pH 600; Spectrum Technologies, Inc. Plainfield, IL, USA). Soil volumetric water content was measured using a moisture sensor (GS1, Decagon Devices, Inc., Washington D.C., USA) at 5 cm depth. Plant root water absorption activity (RWAA) was measured indirectly by monitoring changes in soil water content in each pot. Soil with active root metabolism tends to lose more water when soil water change occurs only through plant transpiration (Stephens & Hering, 2002). We assumed an equal amount of water drainage from pots and surface soil evaporation among the different treatment pots (Allen et al., 1998). Hence, we defined RWAA as the daily amount of water loss during the no watering period. A larger RWAA indicated greater root activity.

Samples (200g) of potting and mineral soils were removed from each pot at the end of the injection period and stored at 4 ° C before analysis. Enzyme analysis was conducted within one week of sampling. Five enzyme activities were determined by fluorometric assays using methylumbelliferone linked substrates, including acid phosphatase (AP), cellobiohydrolase (BC), 1,4-β-glucosidase (BGC), xylosidase (BX) and 1,4-β-N-acetyl glucosaminidase (NAG), which are enzymes that mediate key functions during microbial degradation of soil litter (Chung et al., 2011). AP cleaves phosphoester bonds; BC and BGC decompose cellulose; BX breaks down hemicellulose and NAG degrades chitin (Saiya-Cork, Sinsabaugh & Zak, 2002). The TC and TN contents of air-dried soils were analyzed with a Carlo Erba NS 1500 C/N analyzer (Carlo Erba, Milan, Italy). The hot water extractable carbon (HWC) of the potting and mineral soils, which is a proxy for the amount of labile carbon, was determined following the method described in Nelissen et al. (2012).

### Plant measurements

Photographs were produced every week to record visible changes in plants, and chlorophyll *a* content was measured to monitor physiological changes. To measure chlorophyll *a*, leaves were sampled twice a week from August 20 to September 3 and once a week from September 4 to September 23. The leaves were homogenized by grinding with a mortar and pestle and chlorophyll *a* was extracted using 90% acetone overnight at 4 °C in the dark. A UV/Vis spectrophotometer (Optizen POP, Mecasys Co., Ltd, Daejeon, South Korea) was used to measure chlorophyll *a* concentration (Arnon, 1949).

When the CO_2_ injection period was completed, the plant was carefully dug out and separated into roots and shoots (leaves and stems). Root samples were washed with water and dried at 60 °C for 3 days to obtain dry weights. The fresh weight of the shoots was recorded, and a subsample of fresh shoots was dried to determine water content. To monitor movement and distribution of nutrients, TC, TN and total phosphorus (TP) contents of the plants were measured. The TC and TN contents of the roots and leaves were subjected to combustion analysis with a Carlo Erba NS 1500 C/N analyzer. TP content of leaves was measured using a modified molybdenum blue technique (Murphy & Riley, 1962). The starch content of plant roots was measured using a starch assay kit (K-TSTA, Megazyme International Ireland Co. Ltd., Wicklow, Ireland) as a stress indicator that decreases when plants are under stress (Lake et al., 2012).

### Statistical analysis

Analysis of variance was performed using the MIXED procedure in SAS 9.1 (SAS Institute Inc., Cary, NC, USA) and included concentrations of soil CO_2_ and O_2_, pH, chlorophyll *a* content, and RWAA, while an additional GLM procedure was used to analyze plant parameters (biomass, starch, TC, TN, TP and water content) and soil parameters (TC, TN, HWC contents and enzyme activities). Least square means were used to test for significant differences among treatments at a 5% probability level.

## Results

### Soil gases and pH

**Figure 2:**
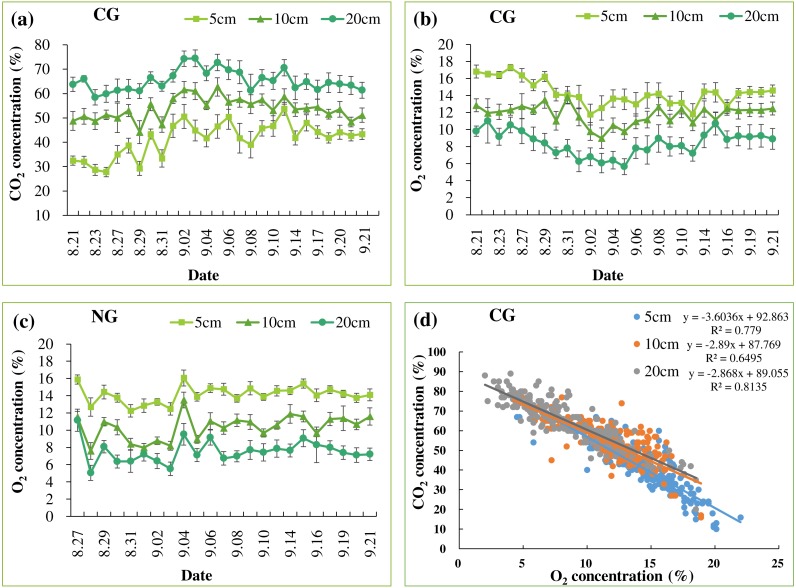
Mean soil CO_2_ (A) and O_2_ (B) concentrations in CG treatment. Mean soil O_2_ concentration in NG treatment (C). The relationship between soil CO_2_ and O_2_ concentrations in CG at each depth (*n* = 256) (D) Bars with the same letters are not significantly different at a 5% level (*n* = 9).

Soil CO_2_ concentrations were significantly higher in the CG treatment than in the NG and BG treatments (Fig. 2, Tables 2 and 3). Due to gas exchange with the atmosphere, we observed a CO_2_ gradient at depths in the CG treatment (Fig. 2A and Table 2). The gas concentrations from locations of nine replicates had small deviations at each depth (Fig. 2), indicating that lateral heterogeneity of gases could be ignored in our small pots. Constant CO_2_ concentrations were maintained throughout the injection period indicating that our incubation system was sufficiently stable to investigate the effects of high soil CO_2_ on plant growth. In the BG and NG treatments, soil CO_2_ concentrations were lower than 1%, which is below the detection threshold (Table 2). These concentrations were comparable with those of field studies that reported <1% CO_2_ in the non-injection plots (0.7–0.9%) measured by high-accuracy gas analyzers (GA5000 and Vaisala 221 GMT probe) (Sharma et al., 2014; Lake et al., 2016a). Soil O_2_ concentrations in the BG treatment were similar at all depths and the same as that at the ambient condition (Table 2). In the NG and CG treatments, soil N_2_ and CO_2_ injections significantly reduced O_2_ concentrations, which were maintained at 7.4–14.3% throughout the injection period (Figs. 2B–2C and Table 2). Soil CO_2_ and O_2_ concentrations were negatively correlated in the CG treatment (Fig. 2D). Soil pH was slightly increased at the end of the injection period compared with the initial pH (Tables 1 and 2), and the CG treatment significantly reduced soil pH compared with pH in the BG and NG treatments (Table 2).

**Table 2:** Mean soil CO_2_ and O_2_ concentrations in different depths and soil pH at each treatment.

Treatments		CO_2_ concentration (%)			O_2_ concentration (%)			pH
	Depth (cm)	5	10	20	5	10	20	
CG		41.3^a^	53.8^a^	65.3^a^	14.3^b^	11.8^b^	8.4^b^	7.4^a^
NG		<1.0^b^	<1.0^b^	<1.0^b^	14.1^b^	10.3^b^	7.5^b^	7.5^a^
BG		<1.0^b^	<1.0^b^	<1.0^b^	21.0^a^	20.9^a^	20.9^a^	7.0^b^

**Notes.**

a,b Different letters indicate significant differences among treatments at a 5% level (*n* = 261).

**Table 3:** Analysis of variance examining the effects of CO_2_ injection on soil parameters.

**Source**	**CO**_**2**_	**O**_**2**_	**pH**	**Chlorophyll *a***	**RWAA**
Date	<0.0001	<0.0001	<0.0001	<0.0001	<0.0001
Depth	<0.0001	<0.0001	–	–	–
Date × Depth	0.0004	<0.0001	–	–	–
Treatment	–	<0.0001	<0.0001	<0.0001	<0.0001
Date × Treatment	–	<0.0001	<0.0001	0.9941	0.0512
Depth × Treatment	–	<0.0001	–	–	–
Date × Depth × Treatment	–	<0.0001	–	–	–

**Source**	**Soil**										
	**Potting**			**Mineral**			**Microbial enzyme activities**				
	**TC**	**TN**	**HWC**	**TC**	**TN**	**HWC**	**AP**	**BC**	**BGC**	**BX**	**NAG**
Treatment	0.6421	0.2532	0.983	0.8781	0.0298	0.0028	0.0042	–^a^	0.2503	0.0174	<0.0001

**Notes.**

a No data.

### Plant parameters during injection

**Figure 3:**
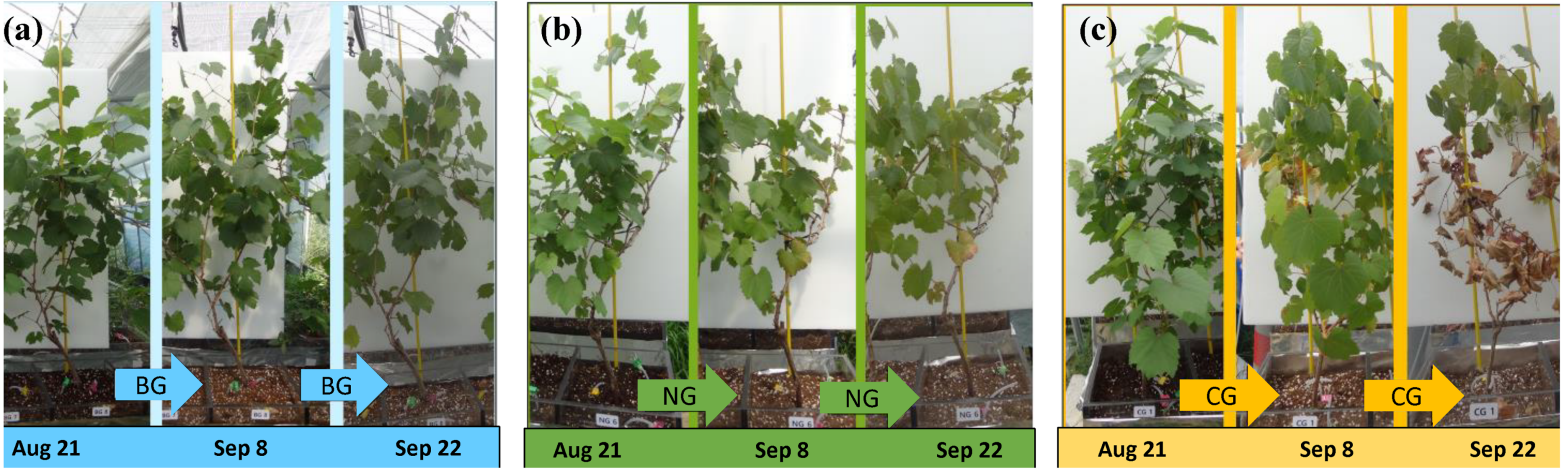
Morphological changes of plants during the experimental period. (A) The photographs of grape in BG (no injection) from left to right were taken on August 21, September 8 and September 22; (B) The photographs of grape in NG (N_2_ injection) from left to right were taken on August 21, September 8 and September 22; (C) The photographs of grape in CG (CO_2_ injection) from left to right were taken on August 21, September 8 and September 22. Photo credit: Wenmei He.

Temporal changes in leaf color and vitality varied by treatment (Fig. 3). In the BG treatment, plant leaves remained healthy and green until the end of the experiment (Fig. 3A). In the NG treatment, plant leaves appeared healthy until September 8 (Fig. 3B); after that, a few leaves at the bottom of the plant turned yellow. In the CG treatment, plant leaves appeared healthy until September 8 and then turned yellow and red. At the end of experiment, the leaves in the CG treatments appeared very dry and showed low vitality (Fig. 3C).

**Figure 4:**
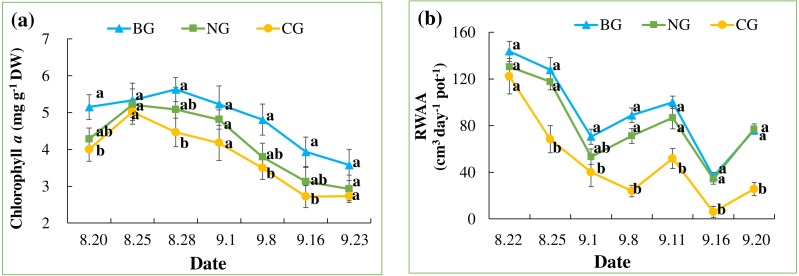
Leaf chlorophyll *a* content (A) and root water absorption activity (RWAA) (B). Each date was compared and results with the same letter are not significantly different at a 5% level (*n* = 9).

In all treatments, chlorophyll *a* content increased quickly until August 25 and decreased slowly thereafter, following the seasonal night temperature drop in all the treatments (Table 3 and Fig. 4A). Treatment effects varied by date and became apparent after the one-week injection. On August 28, September 8, and September 16, the chlorophyll *a* content of leaves in the CG treatment was significantly lower than that in the BG treatment. However, chlorophyll *a* in the NG was not significantly lower than in the BG on any date of measurement.

An overall decreasing pattern in RWAA was observed in all the treatments (Table 3 and Fig. 4B). This could be due to seasonal temperature drop, which leads to slower plant metabolism. Treatment effects became apparent from August 24, when RWAA in the CG treatment was significantly lower than in the BG and NG treatments. This effect continued until the end of the experiment. RWAA was not significantly different between the NG and BG treatments. The effects of high soil CO_2_ on RWAA were observed 4 days earlier than those on chlorophyll *a* content.

### Plant parameters after harvest

After injection, shoot and root biomass were reduced by 19.2% and 59.2%, respectively, in the CG treatment compared with the BG treatment (Table 4). Shoot and root biomass, however, were not changed in the NG compared to the BG. Water content of the shoots (leaves and stems) and starch content of the roots were also significantly lower in the CG treatment, but they were not significantly different between BG and NG treatments. TC content in leaves and roots was similar between BG and CG treatments. TN content was higher in leaves and roots in the CG treatment compared with the BG and NG treatments. The TP content of leaves was lower in the NG and CG treatments than in the BG treatment (Table 4).

**Table 4:** Total shoot and root biomass, shoot water, root starch, TC and TN contents in leaves and roots.

	**Total biomass**	**Shoot biomass**	**Root biomass**	**Shoot water content**	**Root starch**	**TC**		**TN**		**TP**
						**Leaf**	**Root**	**Leaf**	**Root**	**Leaf**
	(g plant^−1^)			(%)		(g kg^−1^)				
**BG**	58.22^a^	34.36^a^	23.87^a^	57.37^a^	16.75^a^	435.78^a^	473.89^a^	17.94^a^	4.80^b^	5.26^a^
**NG**	56.50^a^	35.88^a^	20.62^a^	55.57^a^^,^^b^	17.55^a^	435.82^a^	464.78^b^	15.83^b^	4.43^b^	3.80^b^
**CG**	37.48^b^	27.75^b^	9.73^b^	48.68^b^	2.25^b^	436.42^a^	482.22^a^	19.62^a^	7.89^a^	4.18^b^
*P*	<0.0001	0.0176	<0.0001	0.0754	<0.0001	0.9739	0.0019	0.0970	<0.0001	0.0238

**Notes.**

a,b Different letters indicate significant differences among treatments at a 5% level (*n* = 9).

### Soil parameters after experiments had finished

Soil TC and TN contents did not vary among treatments, while HWC contents in mineral soil were lower in CG than in the BG (Table 5). On the other hand, enzyme activities in potting soil were different among treatments. The activity of AP was lower in the CG and NG than in the BG, while activities of NAG and BX were higher in the NG than in the BG and CG (Table 5). In mineral soils, enzyme activities were too low to be detected.

**Table 5:** TC, TN and HWC contents and microbial enzyme activities in soil.

**Parameters**		**Soils**	**BG**		**NG**		**CG**	
			Potting	Mineral	Potting	Mineral	Potting	Mineral
**Chemical parameters**	**TC**	(g kg^−1^)	250.96^a^	3.38^a^	224.74^a^	3.35^a^	257.76^a^	3.22^a^
	**TN**		4.01^a^	0.68^a^	4.96^a^	0.48^a^^,^^b^	5.17^a^	0.36^b^
	**HWC**		4.62^a^	0.59^a^	4.73^a^	0.57^a^^,^^b^	4.67^a^	0.55^b^
**Microbial enzyme activities**	**AP^c^**	(nmol h^−1^ g^−1^)	200.56^a^	-^d^	114.40^b^	–	86.98^b^	–
	**BC**		–	–	7.93	–	–	–
	**BGC**		83.75^a^	–	75.16^a^	–	50.12^a^	–
	**BX**		5.32^b^	–	10.20^a^	–	5.38^b^	–
	**NAG**		27.30^b^	–	75.22^a^	–	14.63^b^	–

**Notes.**

a, b Different letters indicate significant differences among treatments at a 5% level (*n* = 9).

c AP, BC, BGC, BX and NAG stand for: acid phosphatase, cellobiohydrolase, 1,4-*β*-glucosidase, xylosidase, and 1,4-*β*-N-acetyl glucosaminidase, respectively.

d No data.

## Discussion

The results clearly indicated that injected gases migrated upwards through all the gassed pots. A clear depth gradient of soil CO_2_ was observed in these pots, while lateral heterogeneity in CO_2_ was negligible (Fig. 2). High soil CO_2_ concentration lowered soil O_2_ and pH, consistent with the previous results. The negative relationship between soil CO_2_ and O_2_ concentrations in the CG could be due to O_2_ displacement by injected CO_2_ (Patil, 2012; Lake et al., 2016a). The steeper slope in the linear negative relationship between CO_2_ and O_2_ at 5 cm depth than those at 10 cm and 20 cm (Fig. 2D) implied that, in the shallow surface layer, diffused CO_2_ gas was mixed with atmospheric air (Fig. 2A). These vertical gradients in soil CO_2_ concentration were also observed in other greenhouse and field studies (Krüger et al., 2011; Wu et al., 2014; West et al., 2015; Kim et al., 2018). In addition, our range of CO_2_ concentrations of 41.3–65.3% at 5–20 cm depths (Table 2) was comparable to the levels in previous studies (Beaubien et al., 2008; Al-Traboulsi et al., 2012; Lake et al., 2013; Lake et al., 2016a), and this the possible level of CO_2_ concentration at which CO_2_ from the CCS site would leak and be transported via faults or failure.

The reductions in chlorophyll *a* content, RWAA and total biomass seen with CG treatment and not in the NG treatment indicate that plants were influenced by high soil CO_2_ but not by low O_2_ concentration. Although CO_2_-induced reduction in soil pH in the CG treatment could affect plants, we put little emphasis on the effect of lowered pH. In this experiment, soil pH in the CG treatment (7.0) was lower than in BG and NG (pH = 7.4 on average) (Table 2), but the difference was only 0.4, which is within the optimal range for grape growth. The possible reason for such a slight change in soil pH despite CO_2_ gassing, which could lead to formation of weak acid (H_2_CO_3_) and release of H^+^ (Wei, Maroto-Valer & Steven, 2011; Moonis et al., 2017; Zhao et al., 2017), could be the high buffering capacity of the potting soil and plants’ indirect effects. Our results were consistent with those of Lake et al. (2013), who reported that beetroots were not significantly affected by N_2_ gas treatment at O_2_ concentration of 8.7%. Even in an O_2_-free hydroponic solution, Boru et al. (2003) and Chang & Loomis (1945) did not observe inhibited root growth or function, implying that plants could be resistant to low O_2_ condition (Geigenberger, 2003). Nikolopoulou et al. (2012) reported clear results that grass (*Cynodon dactylon*) root length and biomass were greatly reduced (>50%) under treatment with 40% CO_2_ combined with 20% O_2_, and a similar reduction was observed under treatment with 40% CO_2_ combined with 10% or 2.5% O_2_. We therefore suggest that high soil CO_2_ concentration has negative effects on overall plant metabolism.

We observed the effects of high soil CO_2_ concentration on plants with regard to root function. The reduction in RWAA in the CG treatment was apparent on August 24, 4 days after the start of injection (Fig. 4B). On August 28th, lower chlorophyll *a* content was first observed in the CG treatment (Fig. 4A). Leaf color change became apparent as late as September 8 (Fig. 3C). The series of changes in the CG treatment imply that high soil CO_2_ concentration affected the root system first, which triggered other aboveground changes in the plant. Color changes in the leaves are related to changes in nutrient transport (Chang & Loomis, 1945; Cook, Ward & Wicks, 1983; Boru et al., 2003; Geigenberger, 2003). In our study, the TN content of the roots was higher in the CG than in the BG treatment, while there were similar TN and lower TP contents in the CG plant leaves compared with the BG leaves (Table 4). These results indicated that nitrogen transport from the roots to the leaves was inhibited by high soil CO_2_ concentration.

CO_2_ injection also induced changes in soil HWC content and microbial enzyme activities. Reduced HWC in mineral soil of the CG treatment could be related to the high soil CO_2_ concentration (Table 5). Moonis et al. (2017) reported that soil CO_2_ injection increased exchangeable Al^3+^ of mineral soil, which would be strong bound with dissolved C molecules. The resulting Al^3+^-C complexes could be absorbed on the soil surface, producing reduced water-extractable C. Reduced soil microbial enzyme activity of AP in the CG and NG (Table 5), which is involved in cleaving phosphoester bonds, could be related to the decreased TP contents of the leaves in those treatments (Table 4). The increased enzyme activities of NAG and BX in the NG treatment (Table 5), which are involved in decomposition of hemicellulose and chitin, respectively, might be related to lower soil O_2_ concentration. In a low O_2_ environment, the activity of N_2_-fixation organisms could be enhanced, resulting in increased soil-available N. This change could be beneficial to microbial decomposition processes that are usually limited by the low N content of residues in soil (Rice & Paul, 1972; Halsall & Gibson, 1985; Chen et al., 2011).

**Figure 5:**
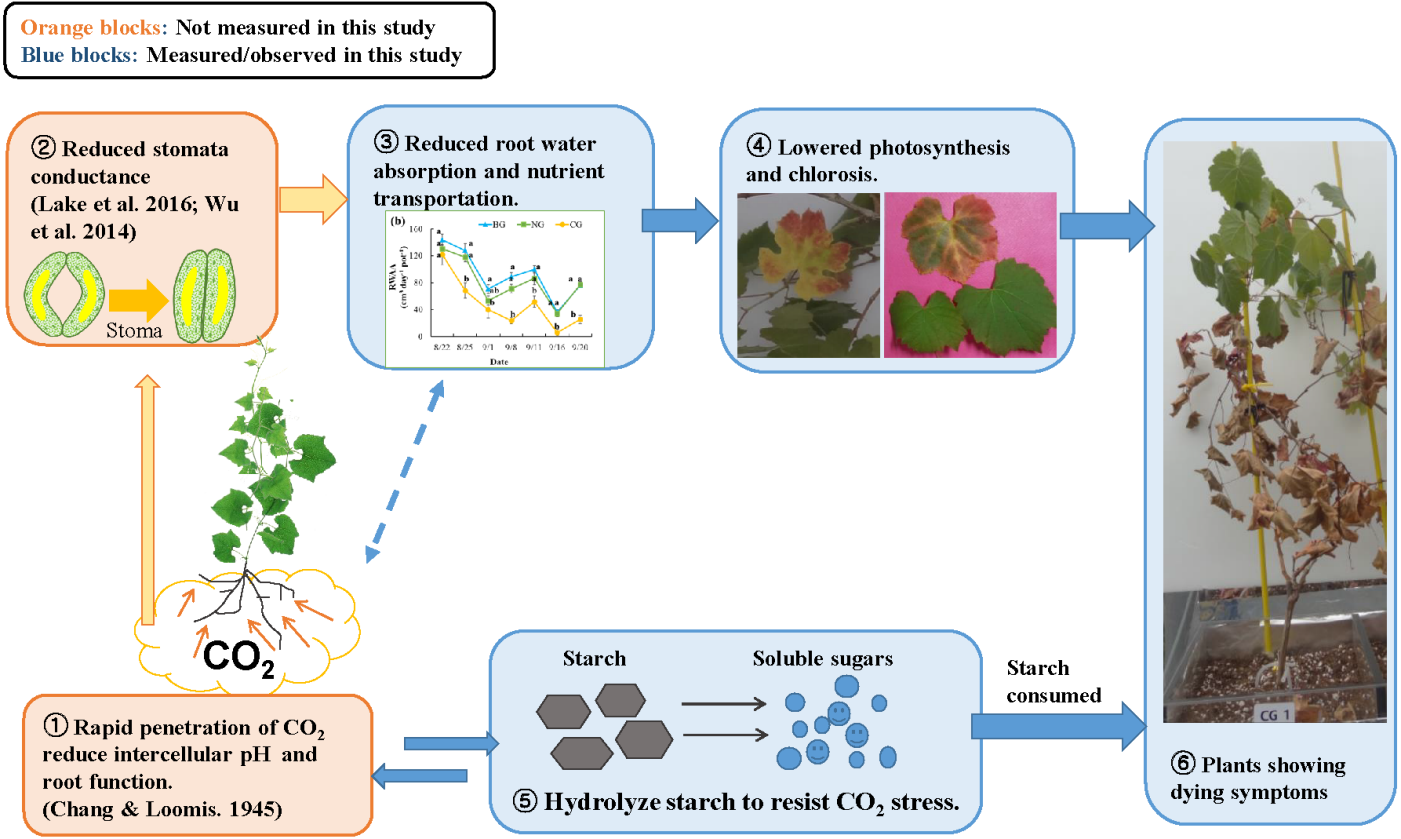
Conceptual model of the response to high soil CO_2_ concentration in plant: involvement of hydraulic reaction and compensational mechanism. Photo credit: Wenmei He.

A mechanism for CO_2_ toxicity to plant root tissue was suggested by earlier studies (Chang & Loomis, 1945; Bown, 1985). High soil CO_2_ concentrations could dramatically change the pH level of root tissues, which could undermine membrane function and osmoregulation of root cells. Damaged root cells would not absorb water sufficiently, and plant stomata would close to prevent water loss (Davies, Kudoyarova & Hartung, 2005). Similarly, Lake et al. (2016b) suggested a signaling mechanism from the root to the leaf: when plant roots suffer CO_2_ stress, they produce hormones such as abscisic acid and send this signal to the leaves to close stomata. As a compensatory mechanism, the plant hydrolyzes starch in the roots and stems into soluble sugars and changes the osmotic potential to increase water absorption (Yang et al., 2001; Mohammadkhani & Heidari, 2008; Naser et al., 2010). In our study, the reduced starch content of roots in the CG treatment (Table 4) could be associated with this compensation response to high soil CO_2_ stress (Nikolopoulou et al., 2012). Based on our observations and extensive literature review, we propose a conceptual diagram of plant response to high soil CO_2_ concentration (Fig. 5). The effects of high soil CO_2_ on plants start from the root cells where intercellular pH is reduced, leading to activation of signaling to the leaf to close the stomata. Due to stomata closure, water and nutrients are not transported from the soil to the leaves, resulting in low photosynthesis. Although plants try to compensate for lower photosynthates by solubilizing starch, they eventually consume stored resources, lose vitally and die.

## Conclusion

Plants can be a useful tool for assessing the potential risk of CO_2_ leakage from CCS sites. We verified that high soil CO_2_ (41.3–65.0%) had negative effects on overall plant metabolism. These negative effects were attributed to high soil CO_2_ concentration and not to low soil O_2_ concentration or to reduced soil pH. Negative effects on root water absorption were observed after CO_2_ injection, and the effects extended to aboveground plant tissues, leading to decreased chlorophyll content and chlorosis.

Results from our study suggest two sensitive indicators for CO_2_ leakage in ecosystem. The first indicator is chlorophyll content which showed a reduction 8 days after CO_2_ injection. Another sensitive indicator was RWAA, which showed reduction 4 days earlier than chlorophyll content. However, as RWAA is difficult to measure in the field, soil water content could be used as an indirect parameter for detecting CO_2_ leak. The inherent heterogeneity of soil water content in space and time could be overcome by establishing long-term baseline data in a given site. Suggested indicators could be utilized to develop a guideline for environmental management of CCS and the surrounding environment. Further research should focus on the responses of different plant species to high soil CO_2_ and the interactions between plants and soil.

##  Supplemental Information

10.7717/peerj.6311/supp-1 Supplemental Information 1 Soil CO_2_ concentration

10.7717/peerj.6311/supp-2 Supplemental Information 2 Soil O_2_ concentration

10.7717/peerj.6311/supp-3 Supplemental Information 3 Chlorophyll *a* contents

10.7717/peerj.6311/supp-4 Supplemental Information 4 Soil moisture

10.7717/peerj.6311/supp-5 Supplemental Information 5 Soil pH

## Additional Information and Declarations

### Data Availability

The following information was supplied regarding data availability: The raw measurements are available in the Supplemental Files.
